# Physical Condition of Horses for Military Purposes

**Published:** 1890-02

**Authors:** George Fleming

**Affiliations:** Principal Veterinary-Surgeon of the Army


### PHYSICAL CONDITION OF HORSES FOR MILITARY PURPOSES.

By George Fleming, Esq., C.B., LL.D., F.R.C.V.S.,
*Principal Veterinary-Surgeon of the Army.*

[Reprinted by permission of the Aldershot Military Society in the *Journal of the Military Service Institution*.]

The subject which I have the privilege and the honor to introduce to your notice to-day, is one of much interest and importance to all who have to do with horses, whether in the Army or out of it; but more especially does it concern those who belong to the mounted portions of our forces; as upon their acquaintance with it, even to the smallest details, will, to a large degree, depend the efficiency and value of their different corps in peace or in war. And nowhere, perhaps, could such a subject be more fittingly introduced and discussed than in this great centre of our military life, where theories can be tested and knowledge practically applied in all that pertains to the successful utilization of troops, whether mounted or dismounted. Here, at Aldershot, we have at all times, and in all seasons, a larger number of horsemen and horses than are located in any other part of the United Kingdom; and the training which they undergo, and the tests and fatigues to which they are exposed, bring them nearer to a realization of what actual warfare is, and what it demands from man and horse, than any other ordeal to which they could be subjected short of war itself.

The value of horses in land wars is so well recognized that it would be sheer waste of time to refer to it; but the value of studying their special aptitudes, their fitness for particular services, and how to obtain and maintain this fitness, is not always sufficiently considered; and when this is the case there is danger that the advantages and benefits which should be derived from them will be only partially available or altogether lost. Whereas, with a good selection of horses for certain services, and judicious management of them, we may derive all that they can yield to man in the performance of the most onerous and severe tasks which may be imposed on them.

How horses can be best and most economically utilized in the Army, is a question which needs the most careful examination; not only because upon the results of it may depend the success of operations in the field, but also because of the increasing difficulty experienced in procuring, in a brief space, a sufficiency of horses to complete army corps, and replace the heavy losses which are generally inevitable among them on active service. It is this question that I venture to bring before you now; and though I feel as if I owe an apology for my temerity in submitting to you views which can have nothing in the way of novelty in them, yet there may be points which will merit discussion, and differing opinions which it may prove beneficial to reconcile, so as to lead to amendment and improvement in horse management.

The physical fitness or condition of horses for military purposes depends upon several factors, the more important of which may be enumerated as:

1. Size and breed of horses for special services.
2. Food, with regard to quantity and quality.
3. Care and management.
4. Amount of work exacted.
5. Manner in which the work is exacted.

### I. SIZE AND BREED OF HORSES FOR SPECIAL SERVICES.

There is not much need to insist upon the necessity for our horse supply being of the best possible quality, and well adapted in every way to the requirements of the different branches of our mounted corps. Our country is pre-eminent for its breeds of light and heavy horses, and we should, therefore, expect that our army horses would excel those of any other nation. I think it is questionable if our superiority in this respect is so marked as it ought to be, or if, with regard to at least one army, it exists at all. Perhaps our draught horses have improved somewhat, but we may entertain doubts whether the cavalry horses have gained much in size and quality during the last quarter of a century. Calling to mind the kind of horses upon which cavalry soldiers were mounted some thirty or forty years ago, one would feel inclined to express a doubt whether they are so good now as they were then. The opinion might be ventured that the modern system of racing has not proved beneficial to cavalry horses; and looking through the different regiments, and comparing the past with the present, we fancy we can see the effect of five-furlong races upon the troop horses of to-day.

Several things conspire to render these horses less suitable than in former times. Race horses were not bred for short races, but for trials of endurance as well as speed, and as it is to the race horse that we generally owe improvement or deterioration in our light horses, as he deteriorates in size and endurance so will our cavalry remounts to a large extent. Again, before the advent of railways, in the days of stage and mail coaches, and when travelers and farmers thronged the roads on horseback, strong, good-paced animals, well adapted for cavalry, were common; now this stamp of steed is almost unknown. Therefore, it is more difficult to obtain horses physically fit for cavalry purposes now than formerly; and though, perhaps, they may show what is termed "Breeding," yet, considering what they have to do on active service, grave doubts may be entertained whether they have sufficient bulk and strength to sustain the trials which they would have to undergo.

It is possible that the steps now taken to improve the lighter breeds of horses throughout the United Kingdom may, in the course of time, give us something like the old-fashioned cavalry horse, fit to sustain successive days of movement under a heavy load and on short rations.

If it be accepted as a fact that our cavalry remounts are, physically, not so good now as they were half, or even a quarter of a century ago, we certainly do not make amends for this by the way we treat them. Many are in the ranks doing hard work before they are five years old; and this is not conducive to their soundness or development. It would be in every way better for them if they were not compelled to undergo the fatigues of mature horses until they were five, or even six, years of age, and perhaps it would be more profitable to the public. Almost every cavalry regiment has a number—some of them a large number—of horses too young for service, physically unfit, in fact; and if they were sent into the field they would probably succumb in a short time. Such regiments, though shown as up to their full establishment in horses on paper, are, therefore, not so; they are deficient in proportion to the number of young and old horses in the ranks. If it could be ordered that no horse should be considered physically fit until he had reached at least five years of age, then the casting period on account of age might be extended to eighteen years. Horses do not reach maturity—that is, the maximum of physical fitness—until they are seven or eight years old. When hard worked too soon, they are often worn out by that time; whereas, when allowed to attain their full growth and strength before they are severely tried, they might be able to perform good service up to even twenty years.

Of course, I am speaking of horses which are fairly well bred, of good conformation, in robust health, vigorous and active. It is all-important that the Army should have such horses. Under-bred animals, whether for draught or saddle, though large enough, are soft in fibre and weak-hearted; they are unable to resist exposure and fatigue, and cannot be trusted on an emergency.

Associated intimately with physical fitness is "Pace." All military horses ought to be smart in movement, and of all the paces which should be found in perfection in them, walking is the one. Good walking, whether in carriage or saddle horses, should be considered as absolutely essential. Though cavalry horses should also trot and gallop well, yet quick walking is as great a desideratum in them. There is much more of this pace on service than of galloping, and it is perhaps the most trying; it is the pace which, according to an old cavalry general, "destroys more horses than do the enemy's bullets."

A horse, then, which may be considered physically fit, if of proper size and conformation for the service he is to perform, should be one which looks bright and lively, walks and trots sound and freely, has a shining coat, hard muscles, and eats, drinks and rests well.

This physical fitness we should find in greatest perfection in our fast-moving horses—in cavalry and horse artillery; for it is upon them that the severest strain is imposed, as they have to move at all paces, and to cover more ground than the heavier horses, which generally move at a uniformly slow pace, and have their loads carried upon wheels. In modern warfare the duties of cavalry will be heavier than in former days, and the brunt of the fatigue will fall upon the horses. Consequently, these horses cannot be too good. Nearly a century ago, a high authority on this branch of the Service, when insisting upon the necessity for cavalry arriving in a fit state on the field of battle, said: "A feeble, delicate horse, shaken by the constant weight it has to carry, and by bad and insufficient food, will be less certain to arrive than a powerful, robust one; or if it does reach the spot, it is exhausted at the very moment when it has need of all its strength. The rapidity of a regiment in the field depends upon the speed of its slowest horses; so that a horse should be able to go at an average gallop."

### II. FOOD: QUANTITY AND QUALITY.

With regard to food in its relations to physical fitness, we all know that this should be sufficient in quantity, and good in quality; but a fact not often recognized, until too late, is that it should also be food to which horses are accustomed.

The quantity should, beyond a certain allowance, be in proportion to the amount of work performed, and perhaps also to the season. In our Army, there is a uniformity in this matter which reduces the question of feeding to the greatest simplicity, though it must be confessed that it is not in harmony with reason or experience. The forage ration is never varied all the year round, no matter how young or how old the animals are, how heavy or how light the work may be (with one or two exceptions to be presently mentioned); and large horses weighing 1,200 or 1,400 lbs. receive the same quantity as those weighing 800 lbs. This ration is, as you are aware:

If in quarters, oats 10 lbs., hay 12 lbs., straw 8 lbs.
If in encampments, oats 12 lbs., hay 12 lbs.
If employed on draught work, 2 lbs. oats extra.

The extra issue is allowed to horses of the Army Service Corps drawing wagons at a trot; and during the Winter months only, for all other draught horses when employed on continuous draught work for a period of at least five hours a day.

An extra issue of 2 lbs. of oats, in addition to the ordinary ration in quarters or in encampments, is allowed to draught horses of the Army Service Corps when these are 16 hands high and upwards.

In Continental armies the allowance of forage is systematically adjusted to the size of all the horses and the nature of their work.

In the German army, for instance, there are four scales of forage rations, the difference between them consisting chiefly in the amount of oats. There is, first, the heavy ration, which consists of 11 lbs. 7¾ ozs.; second, the ration for light cavalry of the Guard, which is 10 lbs. 15 ozs.; third, the medium ration, which is 10 lbs. 11½ ozs.; and fourth, the light ration, which s only 9 lbs. 13½ ozs.

The allowance of hay is 5 lbs. 7½ ozs., and of straw 7 lbs. 10½ ozs.

These rations are issued as follows :

The heavy ration is allowed to horses of general officers, the general staff, adjutants, officers of the War Ministry, cuirassier and guard lancer regiments, military riding school, guard horse artillery and field officers of guard field artillery, all artillery draught horses, those of the gendarmerie and intendance and transport draught horses. Troop horses of the Gardes du Corps Regiment receive at all times 1 lb. 1½ ozs. of oats, and 3 lbs. 4½ ozs. of hay extra.

The second scale is given to the light cavalry of the Guard, guard dragoons and guard hussars.

The medium ration is for the lancer regiments of the line.

The light ration is for all other troops and the horses of officers not specified.

The above are termed the "garrison rations," and are only drawn for horses on the strength ; the three or four extra horses in each squadron, battery and company of transport have to be fed on what can be saved from the forage of the others. It is not compulsory to give the horses their full rations daily, but a portion may be reserved for times when work is heavier than usual.

On the line of march the ration is increased as follows :

Heavy ration to 13 lbs. $3\frac{3}{5}$ ozs. oats.

Light Guard cavalry ration to 11 lbs. 7¾ ozs. oats.

Medium ration to 11 lbs. 4¼ ozs. oats.

Light ration to 10 lbs. 6¼ ozs. oats.

The heavy ration of oats is accompanied by 3 lbs. $4\frac{9}{10}$ ozs. each of hay and straw for feeding ; the other rations having 3 lbs. 4 ozs. of hay, and 3 lbs. 13¼ ozs. of straw for the same purpose, as litter is provided in the billets.

In the field this ration is increased all round by 8¾ ozs. of oats, each horse then carrying what is called "the iron ration of oats"—nearly 14 lbs. the heavy ration, over 12 lbs. the second, 11 lbs. 13 ozs. the third, and 11 lbs. 1 oz. the light ration.

During railway transport each horse is allowed 3 lbs. 4½ ozs. of hay and 2 lbs. 3 ozs. of straw to lay on the floor and ramp of the wagon. If the journey lasts longer than eight hours, 6 lbs. 9 ozs. of hay is allowed extra for every twenty-four hours.

During army corps manœuvres and cavalry division exercises for a period of four weeks, the following rations are allowed :

For cuirassiers or horse artillery draught horses, 12 lbs. 14½ ozs. oats.

For other line cavalry regiments and horse artillery riding horses, 11 lbs. 7¾ ozs. oats.

As on the march, 3 lbs. 4 ozs. of hay and 3lbs. 13¼ ozs. of straw are allowed.

Three-year old remounts at the remount depots receive :

Oats, 6 lbs. 9 ozs.
Hay, 10 lbs. 15 ozs.
Straw, 13 lbs. 2 ozs.

For three or four months of the year they are put on green food, but the transition to and from this is gradual.

In the French Army there is a similar gradation in the scale of forage ration, according to the arm of the service and the kind of work performed. The light cavalry (infantry officers' horses are included in this category) allowance per horse per diem is : Oats about 8¾ lbs., hay 8¾ lbs., straw 5¾ lbs. in garrison ; on the march it is 10 lbs. of oats with the same quantity of hay and straw as in garrison ; at manœuvres, if the horses are in barracks they receive the same ration of oats as in garrison, with 6¾ lbs. of hay and about 9 lbs. of straw, but if in bivouac, then the ration is the same as for the march; while on a war footing it is 10¾ lbs. oats, 6¾ lbs. hay, and 4¾ lbs straw. The line cavalry (the horses of engineer and infantry officers are on this scale) receive in garrison 10 lbs. of oats. 6¾ lbs. of hay, and 9 lbs. of straw ; on the march 11¾ lbs. oats, and 11 lbs. hay and straw ; at manœuvres, if in barracks the ration is the same as in garrison ; if in bivouac it is as on the line of march ; while on a war footing it is nearly 11 lbs. oats, 9 lbs. hay, and 4½ lbs. straw. The reserve cavalry includes the horses of the staff, intendance, staff of artillery and of engineers, and auxiliary transport. The ration in garrison is : Oats 11¼ lbs., hay 9 lbs., straw 9 lbs. ; on the march it is, oats 12 lbs., hay and straw 11 lbs. ; on manœuvres, if in barracks it is the same as in garrison, and if in bivouac the same as on the march ; on a war footing the allowance of oats is 13 lbs., hay 9 lbs., straw 4½ lbs. For artillery horses—draught and saddle—the garrison ration is 10¾ lbs. oats, hay 9 lbs., straw 9 lbs. ; on the march it is 12 lbs. oats, 11 lbs. hay, and the same of straw ; on manœuvres, if in barracks it is the same as in garrison, if in bivouac, the march allowance is given ; the war ration is nearly 13 lbs. oats, 9 lbs. hay, 4½ lbs. straw.

In the Russian Army the daily ration during peace is as follows :

Guard cavalry and artillery, 12 lbs. $7\frac{3}{16}$ ozs. oats, 9 lbs. 1 oz. hay, 3 lbs. 10 ozs. straw.

Line cavalry, artillery and engineers, 9 lbs. 5½ ozs. oats, and the same hay and straw as above.

Transport draught, 7 lbs. $12\frac{11}{20}$ ozs. oats, 18 lbs. 2 ozs. hay.

In the regiments and batteries of the guard, this allowance is given all the year round, but in other corps it is only issued for eleven months, the horses being turned out to grass, and only receive 13 lbs. 9½ ozs. of hay for one month after the manœuvres.

If necessary, barley may be substituted for oats, weight for weight, and hay can replace oats in the proportion of 4 lbs. 8½ ozs. of hay for 3 lbs. $1\frac{4}{5}$ ozs. oats. In war, the above rations are issued with the addition of 3 lbs. 10 ozs. of oats ; and in lieu of straw 4 lbs. 8½ ozs. of hay are issued. Thus, a forage ration of the line cavalry regiment during war is 12 lbs. 15½ ozs. of oats, and 13 lbs. 9½ ozs. of hay.

From this statement you will see that in the great armies of the Continent close attention has been paid to the quantity of forage required in each arm of the service to insure physical fitness in peace and in war ; and you will also gather that in our Army this subject has evidently not received the notice it deserves. Our horses are either underfed while they are performing hard work, or they are overfed when this is light; and if the ration is sufficient for large horses, it must be more than sufficient for small ones. I am certainly of opinion that the whole subject of forage for our troop horses needs investigation; and in view of the fact that the allowance is not properly apportioned between light and heavy horses, or with reference to the work done, and that it is generally inferior to that of other European armies, especially that of Germany, I think the time has arrived when this inquiry should be made. In this inquiry the quality of the forage should not be overlooked; the present contract weight of oats is too low, and should be altered.

### III. CARE AND MANAGEMENT.

With respect to care and management, I need not say much to you, except to state that in direct proportion as these are attended to, so will the physical fitness of horses be ensured. They comprehend so many details, that it is impossible to deal with them in a brief lecture. Grooming, feeding, shoeing, housing, equipping, working, and all the other points comprised in these terms, are of great moment, and need constant attention in order to their being carried out to the advantage of the horses. Given good horses of the proper kind, and food sufficient in quantity and good in quality, their welfare then depends upon those who attend upon them. Only one thing I would like to refer to before leaving this section, and that is the necessity for inculcating kindness in all who have to do with horses. Physical fitness is closely related to mental fitness; for that horses have minds, affections, and memories, no one can deny, and all who have studied them will bear testimony to the effects of ill-treatment and kindness upon them, not only in the performance of their work, but upon their durability. Horses kindly treated will do as much, if not more, service in a given space of time, than those which are harshly used; while they will certainly last very much longer. With unfeeling treatment and bad management, horses can be worn out in a very short period; whereas, under the opposite conditions, their lives might be most usefully prolonged to more than twenty years.

Therefore it is that, in order to assist in ensuring the physical fitness of their troopers, soldiers should be continually reminded of the great importance of kindness to their animal friends and companions. Humanity to animals is a sacred duty imposed upon all of us; to the soldier it is this and something more, for his life may depend upon the reciprocal feeling of affection existing between himself and his steed. In the words of the old cavalry soldier already quoted, "The rider must live only for his horse, which is his legs, his safety, his honor, and his reward." Soldiers should never be deprived of their horses, if possible, if they have taken care of them; but they ought to be encouraged to form a friendship with them, or learn as much as they can of the care and management needed to keep their horses in the best condition for work, and how to preserve them from injury.

### IV. AMOUNT OF WORK EXACTED.

The amount of work which can be safely exacted from horses depends so much upon the points I have already specified that nothing definite can be said with regard to it. Army horses are more exposed to extremes of toil than other horses, and during active operations hardship is usually added to fatigue—hunger and thirst, exposure to inclement weather, and often more or less serious injuries being superadded. It is under these conditions that the physical fitness of horses is most seriously put to the test; and well it is if this enables them to undergo them without breaking down, or becoming seriously impaired in efficiency at a perilous crisis. It only too often happens that there is no choice, during war operations, between moderate, heavy, and excessive work, and, therefore, no rules can be laid down which might be made applicable. What is comparatively easy work for horses in good condition, during favorable weather, and in a country well adapted for movement, would be the opposite for weak horses in an inclement season, and in a heavy country.

### V. THE MANNER IN WHICH THE WORK IS EXACTED.

Of course, everything should be done to guard against physical unfitness, no matter how severe the duty may be; and it is here that the skill and attention of officers and men are perhaps rendered most conspicuous and beneficial. In performing exactly the same amount of work under the same conditions, one regiment or battery will have its horses in a good state during the whole time, while another will be nearly broken down. This will greatly, if not altogether, depend upon the manner in which the work is exacted, and the amount of care taken of the horses. It is well known that there are horsemen who will get more work out of their horses, and at less expense to them, than others; and that a horse which, with judicious management, will perform a journey of many miles, can be exhausted in few miles by unwisely urging him. It is not always the amount of work, but the manner in which it is performed, that does the damage.

Therefore it is that, in order to keep horses physically fit for their work, the utmost vigilance, knowledge, and forethought are imperatively necessary on the part of those who are responsible; and the task is not a light one in the field. At no time was it an easy one, and during active operations in the future it is to be apprehended that it will be more onerous than ever it was; it will certainly be no less exacting than it was during the Continental wars in the early part of this century, when a light cavalry general enumerated, among other requirements of a good cavalry officer, "readiness in judging of the state of health of men and horses; a knowledge of prompt remedies applicable in certain cases; a daily and careful inspection of the saddlery, and detection of the repairs required in it; provision of everything which may be useful to man and horse, without over-weighting the latter; inspection of the equipment and of the repairs required in it; intelligent packing of kit; regularity of pace on the march; good choice of ground for camping, and continuous supervision of everything which may affect the health of the horse there; the teaching of the means by which the services of a farrier may be dispensed with for the time being; * * * watchfulness to prevent useless expenditure of the strength of his horses; to give, in all circumstances, a personal example, and to give it all the more steadfastly as the situations are more trying and difficult."

For horses which have to move quickly over broken ground, and perhaps at the end of a long march, the greatest consideration is required to keep them ready for all emergencies; and it is to these horses that the foregoing remarks more especially apply. While the greatest attention is being paid to render the fire of artillery and infantry more rapid and deadly, and more extended, are we taking as much care to improve the mobility, the celerity of our cavalry in proportion, so as to place it on a more equal footing with these now formidable arms? I fear the answer must be in the negative; for while our horses have certainly not improved in quality, as cavalry horses, from what they were half a century ago, when fire-arms of precision were not known, the loads carried by these horses have not been much, if anything, lessened. Not only have the arms of precision rendered it necessary that physical fitness in endurance and speed should be more than ever considered in our quick-moving horses; but the electric telegraph, telephone, and visual signaling, have all concurred in demanding greater rapidity of movement from them, if cavalry is to be a potent factor in warfare. In connection with this point, the question of the weight carried by cavalry horses is of the most vital importance. There can be no doubt whatever, that for the work which cavalry should do effectively in the field, the loads they carry are far too great. So far as I can learn, the estimated weight that a light cavalry horse should carry is more than 19 stone, showing the rider to weigh 10 stone; in heavy cavalry, the soldier weighing 12 stone, the horse carries more than 21 stone, and this without even a day's forage, which will add at least another stone; and if the weather be wet, saturation will impose yet another stone or two more. Celerity under such enormous loads is impossible; and when to these we have bad weather and scanty or bad forage, with long marches, it is evident that the destruction of the best horses is a matter of a few days; exhaustion must be rapid, and sore backs and crippled limbs inevitable.

There would appear to be a tendency to heap weight on horses' backs, even in mounted corps with carriages, as in the artillery and transport.

In the cavalry, the soldier carries on him 28 lbs. 3¼ oz.; the horse equipment 74 lbs. 3½ ozs.; in wallets 6 lbs. 14¾ ozs.; in front of saddle 8 lbs. 1¾ ozs.; behind saddle 12 lbs. 13½ ozs.

In the Royal Horse Artillery, the soldier carries 28 lbs. 7¼ ozs.; the horse (including harness) 98 lbs. 14½ ozs.; in wallets 8 lbs. 3¾ ozs.; in front of saddle 8 lbs. 1¾ ozs.

In Slow Draught Corps (Royal Artillery, Royal Engineers, and Army Service Corps), articles on driver weigh 18 lbs. 8 ozs.; harness complete 80 lbs. 8 ozs.; articles on saddle 26 lbs. 8 ozs.; articles in wallets 4 lbs. 8 ozs.

So that in the Royal Horse Artillery the near side horse carries 144 lbs., which, with the weight of the driver (12 stone) amounts to 22 stone, while in a field battery it is 21 stone; and these horses have to draw from 8 to 10 cwt. besides!

These terrible loads could surely be reduced, even if we must consider them as minimum—for it must be remembered there are many men in cavalry who weigh much more than 12 stone. I have seen men in light cavalry, who, in stable dress, weighed 16 and 17 stone. It does seem strange, indeed, that in corps such as artillery and transport, the greater part of the killing load should not be transferred from the horses' backs to the wagons. An ounce on wheels is a pound on the saddle.

And surely the cavalry loads can be lightened considerably. Is it absolutely necessary that all the articles enumerated in the equipment list be carried on the saddle?

Wheel or pack saddle carriages should be employed to convey everything but what is vitally necessary for man and horse. It is astonishing how little is needed, if men are trained to require little. De Brack, who should have known what was necessary, says: "A valise for light cavalry which can hold more than a couple of shirts, a holdall, and under its flap a pair of boots, is not only useless, but really a danger. The fewer articles a soldier has the greater care he takes of them, the cleaner he is, and the more ready, too. The chasseurs of the Imperial Guard went through the whole of the Russian campaign before my eyes, with a pelisse and a single pair of cloth Hungarian pantaloons." And he adds: "The art of carrying the kit lies in three things—1st, to take only what is indispensable; 2d, to distribute the weight equally, fatiguing the horse as little as possible, and not chafing him; 3d, to afford the rider the greatest possible facility in managing his horse, and to derive the greatest advantage from its powers. The art of packing is three-fourths of the duties of a cavalry soldier in the field."

The work of a cavalry horse is more like that of a hunter than any other description of horse; and we know what a hunter can do and how he must be treated in order to keep him physically fit. We also know something as to the price of a hunter able to carry 18 stone safely, and if he does two days' hunting in seven it is usually considered enough; in the meantime, he receives such treatment in the way of housing, feeding, grooming, and other attentions, as the poor troop horse never knows at any time.

It is urgently required that the loads on army horses' backs be diminished to the very lowest possible weight. At no time should it exceed 18 stone, even with the best horses this country can produce; and that army which can muster a lightly equipped cavalry will certainly have a great advantage over one whose horses are heavier laden.

How reduction of weight is to be effected it is scarcely in my province to say, though I might urge that on the saddle and on the soldier little more should be carried than what is needed for actual conflict, and these articles should be of the very best. Everything else necessary should be carried on wheels or pack saddle.

It may be interesting to glance at the weights carried by cavalry horses in the armies of other countries, and compare them with those of our own.

FRANCE.

According to the Aide Mémoire of the Staff (edition 1888), the following are the average weights, including the weight of the rider:

| | | | | |
|---|---|---|---|---|
| Light cavalry . . . . . . . . . . . | 19 stone | 1 lb. | = | 266 lbs. |
| Dragoons . . . . . . . . . . . . . | 20 " | 2 " | = | 282 " |
| Cuirassiers . . . . . . . . . . . . | 23 " | 10 " | = | 332 " |

It is added that these weights are increased by 1 stone 8 lbs. after exposure to rain.

A more recent article[1] in the *Revue de Cavalerie* (May, 1889), gives the following as the result of official trials in several regiments:

| | | | | |
|---|---|---|---|---|
| Light cavalry . . . . . . . . . . | 17 stone | 11 lbs. | = | 249 lbs. |
| Dragoons . . . . . . . . . . . . . | 18 " | 10 " | = | 262 " |
| Cuirassiers . . . . . . . . . . . . | 21 " | 6 " | = | 300 " |

These weights were made up as follows:

| | Average weight of man. | Average weight of saddlery, etc. |
|---|---|---|
| Light cavalry . . . . . . . . | 9 stone 6 lbs. | 8 stone 5 lbs. |
| Dragoons . . . . . . . . . . . | 10 " 1 " | 8 " 9 " |
| Cuirassiers . . . . . . . . . . | 11 " 9 " | 9 " 11 " |

I may incidentally mention that the French light cavalry horses toward the earlier part of this century carried 112 to 115 kilograms, equal to 249 to 254 lbs., or 17 stone 11 lbs. to 18 stone 2 lbs.; being an average of 17 stone 12 lbs.

ITALY.

Colonel Slade[2] gave the following as the weight of Italian cavalrymen with arms and accoutrements (forage not included):

| | | | | |
|---|---|---|---|---|
| Hussars . . . . . . . . . . . . . . | 16 stone | 8 lbs. | = | 232 lbs. |
| Lancers . . . . . . . . . . . . . . | 17 " | 4 " | = | 242 " |

This month he forwarded the following additional information:

LIGHT CAVALRY.

| | | |
|---|---|---|
| Average weight of man . . . . . . . . . . | 10 stone | 5½ lbs. |
| " " saddle complete . . . . | 4 " | 6½ " |
| " " carbine and sword . . . | | 13 " |
| | 15 " | 11 " |

LANCERS.

| | | |
|---|---|---|
| Weight of man . . . . . . . . . . . . . . . | 11 stone | 4½ lbs. |
| " saddle complete . . . . . . . . | 4 " | 6½ " |
| " sword, lance and carbine . . . . | 1 " | 3½ " |
| | 17 " | 0½ " |

In addition to the above the horse carries 12 lbs. of hay and 8 lbs. of corn.

[1] Entitled "Trop Lourd."

[2] In despatch No. 51 of 1888.

Colonel Slade adds that the weights given in his report, No. 51, were taken from personal observations, as he had six men of each branch of the cavalry weighed, and struck an average. They differed a little from the above, which he got from the War Office, and on one point in particular, he disagreed, viz., the difference between the individual weight of a light cavalryman and a lancer, as there is certainly not more than 7 lbs., whereas they show it to be 13 lbs.

### BELGIUM.

Particulars of the new light saddle and equipment adopted for the Belgian cavalry will be found in War Office paper $^{0}\frac{140}{306}$ with enclosures.

This saddle, designed by General Courtin, when fully equipped for the field, weighs :

3 stone 1 lb.

The weight of corn for the horse and the rider's rations are included.

### GERMANY.

Cuirassier's horse (marching order)

| | | | |
|---|---|---|---|
| carries . . . . . . | 23 stone | 0.388 lbs. | = 322 lbs. |
| Man, average . . . | 13 " | 5 " | |
| Saddlery and kit . . | 6 " | 6.002 " | |
| Clothing and arms . | 3 " | 3.386 " | |

Hussar's horse (marching order)

| | | | |
|---|---|---|---|
| carries . . . . . . | 18 " | 5.554 " | = 252 lbs. |
| Man . . . . . . . . . | 10 " | 3 " | |
| Saddlery and kit . . | 6 " | 7.630 " | |
| Clothing and arms . | 1 " | 1.8924 " | |

### NETHERLANDS.

Cavalry horse (marching order)

| | | | |
|---|---|---|---|
| carries . . . . . . | 17 stone | 12.8 lbs. | = 250 lbs. |
| Man . . . . . . . . . | 10 " | 3 " | |
| Saddlery and kit . . | 5 " | 4.8 " | |
| Clothing and arms . | 2 " | 5 " | |

### SWITZERLAND.

Cavalry horse (marching order)

| | | | |
|---|---|---|---|
| carries . . . . . . | 17 stone | 5.66 lbs. | = 243 lbs. |
| Man . . . . . . . . | 10 " | 3 " | |
| Saddlery and kit . . | 5 " | 0.05 " | |
| Clothing and arms . | 2 " | 2.61 " | |

### SWEDEN.

Cavalry horse (marching order)

| | | | |
|---|---|---|---|
| carries . . . . . . | 19 stone | 8.17 lbs. | = 274 lbs. |
| Man (say) . . . . . | 10 " | 3 " | |
| Saddlery and kit, Clothing and arms (bracketed) | 9 " | 5.17 " | |

RUSSIA.

The average weight carried by the cavalry horse is 18 to 20 stone, as follows;

| | | |
|---|---|---|
| Saddlery complete with valise and great coat | 83½ | lbs. |
| Clothing and boots | 8 | " |
| Linen | 1¾ | " |
| Carbine, bayonet and cartridge pouch | 11 | " |
| 40 rounds ammunition | 3½ | " |
| Weight of man | 144½ to 180½ | " |

AUSTRIA.

SERVICE MARCHING ORDER.

| | |
|---|---|
| Dragoons, Uhlans | 21 stone 3 lbs. |
| Hussars | 20 " 13 " |

The weight of the rider is in each case taken at 11 stone.

I have now concluded my observations on the physical condition or fitness of army horses, but feel that I can have done the subject only scant justice. It is far too extensive for one lecture, but, nevertheless, I trust what I have ventured to bring before you relating to it may be amplified by the remarks of those of you who are in a better position to deal with it than I can pretend to do.

[Ed. Note. UNITED STATES ARMY.

| | | | |
|---|---|---|---|
| Cavalry horse (marching order) carries | 226 | lbs. | |
| Man, average | 145 | " | |
| Saddlery and kit | 37 | " | 1 oz. |
| Clothing | 24 | " | |
| Arms and ammunition | 20 | " | |

